# Adenoviral Transfer of Human Aquaporin-8 Gene to Mouse Liver Improves Ammonia-Derived Ureagenesis

**DOI:** 10.3390/cells12111535

**Published:** 2023-06-02

**Authors:** Alejo M. Capiglioni, María C. Capitani, Julieta Marrone, Raúl A. Marinelli

**Affiliations:** Instituto de Fisiología Experimental, Consejo Nacional de Investigaciones Científicas y Técnicas, Facultad de Ciencias Bioquímicas y Farmacéuticas, Universidad Nacional de Rosario, Rosario 2000, Argentina

**Keywords:** aquaporin-8, ammonia, mitochondria, ureagenesis

## Abstract

We previously reported that, in cultured hepatocytes, mitochondrial aquaporin-8 (AQP8) channels facilitate the conversion of ammonia to urea and that the expression of human AQP8 (hAQP8) enhances ammonia-derived ureagenesis. In this study, we evaluated whether hepatic gene transfer of hAQP8 improves detoxification of ammonia to urea in normal mice as well as in mice with impaired hepatocyte ammonia metabolism. A recombinant adenoviral (Ad) vector encoding hAQP8, AdhAQP8, or a control Ad vector was administered via retrograde infusion into the bile duct of the mice. Hepatocyte mitochondrial expression of hAQP8 was confirmed using confocal immunofluorescence and immunoblotting. The normal hAQP8-transduced mice showed decreased plasma ammonia and increased liver urea. Enhanced ureagenesis was confirmed via the NMR studies assessing the synthesis of ^15^N-labeled urea from ^15^N-labeled ammonia. In separate experiments, we made use of the model hepatotoxic agent, thioacetamide, to induce defective hepatic metabolism of ammonia in mice. The adenovirus-mediated mitochondrial expression of hAQP8 was able to restore normal ammonemia and ureagenesis in the liver of the mice. Our data suggest that hAQP8 gene transfer to mouse liver improves detoxification of ammonia to urea. This finding could help better understand and treat disorders with defective hepatic ammonia metabolism.

## 1. Introduction

Ureagenesis is the main pathway for handling ammonia derived from amino acids and protein breakdown/turnover. Thus, efficient ammonia detoxification is essential for the prevention of hyperammonemia and hepatic encephalopathy [1]. Deleterious effects of high ammonia levels spotlight the need to elucidate new strategies for the treatment of this issue.

Urea production is located predominantly in periportal hepatocytes, where the enzyme carbamoyl-phosphate synthetase 1 (CPS1) is the first and the rate-limiting step of the cycle [2]. Ammonia has to permeate the mitochondrial membrane prior to the first step of metabolization [1]. Therefore, mitochondrial ammonia uptake is a possible metabolic target to improve the outcome of the urea pathway.

Aquaporin-8 (AQP8) is a member of the aquaporin family that exhibits permeability, in addition to water, to small neutral solutes, such as ammonia [3,4]. In hepatocytes, AQP8 could be found as a glycosylated protein, which is expressed in canaliculi and cytoplasmic vesicles [5], and in a non-glycosylated form (28 kDa) expressed in the inner mitochondrial membrane [6]. Liver mitochondrial AQP8 has been found to be correlated with mitochondrial ammonia permeability [7]. Thus, the knockdown of mitochondrial AQP8 expression in culture hepatocytes causes impaired ammonia-derived ureagenesis [8]. In addition, AQP8 overexpression in cultured hepatocytes enhances ureagenesis capacity in response to ammonia challenge [9].

On the other hand, a downregulation of hepatocyte AQP8 has been found in studies using hepatotoxic agents with an impact on ureagenesis, suggesting a plausible mechanistic involvement of AQP8 in ammonia-derived defective ureagenesis [10,11].

In this study, we studied whether the transfer of the human AQP8 gene to mouse liver could improve the outcome of the urea cycle after ammonia challenge. Furthermore, we evaluated whether AQP8 expression could function as an enhancer of ammonia detoxification in an animal model of impaired ureagenesis.

## 2. Materials and Methods

### 2.1. Chemicals

Sucrose was obtained from MP Biomedicals. Thioacetamide (TAA), formamide, phenylmethylsulfonyl fluoride (PMSF), and ammonium acetate were purchased from Sigma-Aldrich (St. Louis, MO, USA). 4′,6-diamidino-2-phenylindole (DAPI) was obtained from Invitrogen (Waltham, MA, USA). Leupeptin was obtained from Chemicon Millipore (St. Louis, MO, USA). For the immunoblotting materials, Polyscreen PVDF transfer membrane was from Perkin Elmer Life and Analytical Sciences (Waltham, MA, USA); Pierce ECL Western blot analysis substrate was from Thermo Fisher Scientific (Waltham, MA USA); and Hyperfilm MP was from Cytiva (Amersham, UK)

### 2.2. Animals

The animals in this study received humane care according to the Guide for the Care and Use of Laboratory Animals (National Institutes of Health, Bethesda, MD, USA). The protocols were approved by the Comisión Institucional para el Cuidado y Uso de Animales de Laboratorio (CICUAL), Facultad de Ciencias Bioquímicas y Farmacéuticas, UNR (Res. 496/2018). The experiments were conducted with male seven-week-old C57BL/6 mice that were divided randomly into experimental groups, as indicated below.

### 2.3. Adenoviral Administration

We made use of AdhAQP8, a bicistronic recombinant adenovector serotype 5, which is a replication-deficient vector encoding for hAQP8 and enhanced green fluorescent protein (EGFP) [12], and an adenovector serotype 5, which is a replication-deficient vector encoding only EGFP (control adenovector). The mice were anesthetized with isoflurane; the induction was conducted in a saturated chamber with isoflurane 4% under oxygen at a flow rate of 0.8 L/min until the loss of the righting reflex. After induction, the mice were moved to a thermostated platform, and anesthesia was maintained with isoflurane 2% under oxygen at the same flow rate through a facemask during the procedure. A 2 cm midline laparotomy was performed just below the xyphoid through the alba line to expose the duodenum. The cystic duct was clamped and, then, a bile duct catheterization was carried out by placing a PE-10 polyethylene catheter through the Vater papilla. After that, 0.1 mL of a solution containing 3 × 10^9^ plaque-forming units of the AdhAQP8 or control adenovector was administered over a period of 3 min, and then, the catheter was kept in place for 15 min to stop backflow. After the adenovector administration, the catheter was gently removed, and the abdominal incision was sutured. The mice were monitored every 24 h for 72 h until the experiments were started. The ammonia challenge studies were carried out via a single intraperitoneal injection with ammonium acetate at 25 mg/Kg of body weight in a PBS solution. After 15 min, blood was collected via cardiac puncture and euthanasia was performed via exsanguination.

### 2.4. Liver Urea Determination

Urea concentration was determined from total liver homogenates. Briefly, samples were prepared from liver homogenates in 0.3 M cold sucrose, which was supplemented with protease inhibitors (Leupeptin at 0.1 mM and PMSF at 0.1 mM), at a 1:4 ratio using 10 up-and-down strokes with a Glass-Teflon homogenizer with a pestle rotation of 1000 rpm. The samples were centrifuged at 1000× *g* at 4 °C for 15 min, and the supernatant, which was free of nuclei and cellular debris, was used for the determination (Urea Assay Kit, Abnova, Taipei, Taiwan) [13].

### 2.5. Plasma Ammonia Determination

Plasma ammonia levels were determined after 15 min of ammonium acetate injection using an “Ammonia kit” (Wiener lab, Rosario, Argentina). Briefly, the kit used the glutamate dehydrogenase enzyme, α-ketoglutarate and NADPH to measure the absorbance change in extinction at 340 nm as a directly proportional value to the ammonia concentration in the samples.

### 2.6. Assessment of ^15^N-Labeled Urea Using NMR Spectroscopy

^15^N-labeled urea for ureagenesis was assessed from total liver homogenates. The total liver homogenates were prepared as described above, and the supernatants were lyophilized and resuspended in deuterated DMSO. ^15^N-labeled urea was detected using nuclear magnetic resonance (NMR) assessing the peak appearance at about 76.7 ppm and using ^15^N natural abundance formamide (0.365%) as an internal standard with a peak at about 113.3 ppm. The operating settings and procedures for NMR were performed as previously described [8]. The differences between the groups were quantified by integrating the area under the ^15^N-labeled urea peak and relativized to the corresponding internal standard.

### 2.7. Preparation of Mitochondrial Fraction

Liver post-nuclear supernatants, which were prepared as described above, were centrifuged at 6000× *g* at 4 °C for 10 min to obtain mitochondrial pellets. Thereafter, the pellets were washed twice with sucrose supplemented with protease inhibitors and stored as mitochondrial fractions.

### 2.8. Immunoblotting

The mitochondrial fractions were treated with a sample buffer (50 mM Tris, pH 6.8, 10% glycerol, 1.3% SDS, and 100 µM DTT) and heated at 90 °C for 5 min. The samples were loaded in a 12% SDS-polyacrylamide gel, separated by electrophoresis, and then transferred to a PVDF membrane. After 1 h of incubation with a blocking buffer (PBS, 3% Tween, and 3% albumin), the membrane was washed and incubated overnight with the primary antibodies at 4 °C. The primary antibodies used were rabbit monoclonal anti-AQP8 antibody [EPR8397] (ab133667) (Abcam, Cambridge, UK) (1 μg/mL), ornithine transcarbamylase (AV41766) (OTC) (1 μg/mL), and carbamoyl phosphate synthetase 1 (AV45689) (CPS1) (0.5 μg/mL) (Sigma-Aldrich). Rabbit antibody (ab28172) (Abcam) against prohibitin (0.5 μg/mL), an inner mitochondrial membrane protein, was also used. After that, the blots were repeatedly washed and incubated with a secondary antibody against rabbit IgG conjugated to horseradish peroxidase for 1 h. Finally, proteins were detected using a chemiluminescent kit (ECL Pierce, Thermo Fisher Scientific) and via the exposure of autoradiographic films (Cytiva), and the protein bands were analyzed based on densitometry in Fiji (ImageJ) v. 2.9.0/1.53t.

### 2.9. Confocal Immunofluorescence

OCT-embedded liver sections (4 μm thick) were mounted on a positively charged slide (SuperFrost^®^ Plus, Menzel-Gläser; Thermo-Scientific, Waltham, MA, USA), incubated for 15 min with a blocking buffer (PBS; 0.2% Triton X-100; and 3% bovine serum albumin), and then incubated overnight at 4 °C with rabbit monoclonal AQP8 antibodies (1/100 dilution). Then, the samples were washed and incubated with a secondary Alexa Fluor 647-conjugated goat anti-rabbit secondary antibody (Molecular Probes, Eugene, OR, USA) for 1 h. After washing the samples, nuclei were stained with DAPI (50 μM) for 10 min, and the coverslips were mounted with ProLong Gold. The samples were visualized using confocal microscopy (Nikon C1 Plus confocal microscope mounted on an Eclipse TE-2000-E2 inverted microscope), and the same microscope settings were used for imaging acquisition. No autofluorescence signals were detected in the samples incubated only with the primary or secondary antibody. In order to analyze the collected images, the same contrast/brightness adjustments were applied to every image within the set using the Fiji (ImageJ) software v. 2.9.0/1.53t. Transduction efficiency was calculated as previously described [14].

### 2.10. TAA Treatment

At 24 h after adenovector administration, the mice were injected intraperitoneally with a single dose of TAA at 25 mg/kg of body weight in a PBS solution, or vehicle. The samples for functional and immunoblotting analysis were obtained 48 h after the TAA injection.

### 2.11. Statistical Analysis

The experimental data are expressed as means ± SEM. Significance was determined using Student’s *t*-test or one-way ANOVA with Tukey’s test. *p* < 0.05 was considered statistically significant.

## 3. Results and Discussion

The main finding of this study relates to the functional importance of mitochondrially expressed hAQP8 channels in hepatic ammonia-derived ureagenesis in mice. We provide experimental evidence that the in vivo hepatic adenoviral delivery of hAQP8 gene (i) induced mitochondrial expression of hAQP8; (ii) enhanced conversion of hepatic ammonia to urea in normal mice; and (iii) improved ammonia detoxification in a mouse model of defective hepatic ammonia metabolism.

### 3.1. Hepatocyte Mitochondrial Expression of hAQP8 in AdhAQP8-Transduced Mice

**Figure 1:**
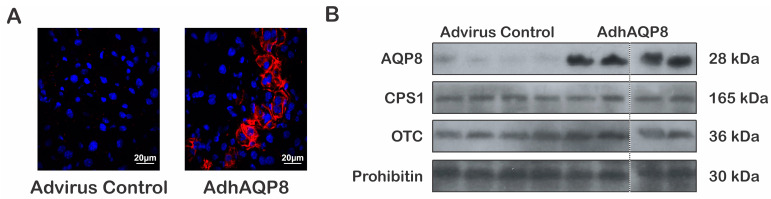
Hepatocyte expression of hAQP8 in mice transduced with AdhAQP8. (**A**) Confocal immunofluorescence microscopy for hAQP8 in liver tissue of mice transduced with control or AdhAQP8 vector (red). In accordance with the reported localization of endogenous AQP8 in hepatocytes, hAQP8 immunoreactivity was observed intracellularly as well as on cell surface. Note that with the confocal settings used, endogenous AQP8 was almost undetected. Nuclei were stained with DAPI (blue). As detailed in the results section, roughly 20% of total hepatocytes were consistently transduced. (**B**) Immunoblotting for AQP8 and mitochondrial urea cycle enzymes in liver mitochondrial fraction. A 28 kDa immunoreactive band corresponding to endogenous mitochondrial mouse AQP8 (weak staining) and hAQP8 is observed. The AQP8 antibody used is able to detect both mouse and human isoforms but with a higher affinity for the latter. Carbamoyl-phosphate synthetase 1 (CPS1) and ornithine transcarbamylase (OTC). Each lane was loaded with 25 μg of protein. Prohibitin, an inner mitochondrial membrane marker, is shown as the control for equal protein loading.

The mice were transduced with an adenovector encoding hAQP8, AdhAQP8, or a control adenovector (see Materials and Methods for details). After 72 h, multiple groups of hAQP8-transduced hepatocytes were observed via confocal immunofluorescence (Figure 1A). In agreement with the known localization of endogenous AQP8 in hepatocytes [6], hAQP8 immunoreactivity was observed intracellularly as well as on cell surface (Figure 1A). We used an antibody that detects both mouse and human isoforms of AQP8, but with a higher affinity for the latter, as reported by the manufacturer. Thus, at the confocal settings used, endogenous AQP8 was not evident. The mitochondrial expression of hAQP8 was confirmed using liver subcellular fractionation and immunoblotting. Figure 1B shows that a band of expected size at around 28 kDa, which is strongly detected in the hepatic mitochondria from the AdhAQP8-transduced mice. The expression of the mitochondrial urea cycle enzymes, CPS1 and OTC, was not altered (Figure 1B). The results (means ± SEM) were as follows: CPS1 (arbitrary units/prohibitin arbitrary units): Advirus control: 5.7 ± 0.7 versus AdhAQP8: 7.2 ± 1.0, and OTC (arbitrary units/prohibitin arbitrary units): Advirus control: 10.4 ± 1.3 versus AdhAQP8: 10.8 ± 0.5.

Based on the immunostaining, about 20% of hepatocytes were transduced with AdhAQP8. This transduction efficiency via a retrograde adenovector infusion agrees quite well with previous reports from our laboratory [14] and others [15]. 

Ureagenesis is a metabolism that is zone specific in the hepatic acinus. Urea production takes place primarily in periportal hepatocytes, which express complete pathway enzymes as well as AQP8 with a similar expression profile [16]. It is important to mention that hepatocytes in the periportal area are the ones that are predominantly transduced after the retrograde biliary infusion of adenovirus [14,17]. Thus, intrabiliary infusion is adequate for the expression of hAQP8 in hepatocytes without causing any significant adverse effects, as previously reported [14,18,19]. In addition, it allows adenovirus delivery to hepatocytes with minimal leakage out of the liver due to anatomical limitations of the biliary tract [14]; furthermore, excess adenovirus is immediately delivered to the duodenum and excreted in feces. Therefore, repetitive transgenic hepatic expression could be achieved via intrabiliary infusion without immunosuppression [17]. Whether hAQP8 is stable for a long time after adenovector administration remains to be elucidated.

### 3.2. Ammonia-Derived Ureagenesis in AdhAQP8-Transduced Mice

**Figure 2:**
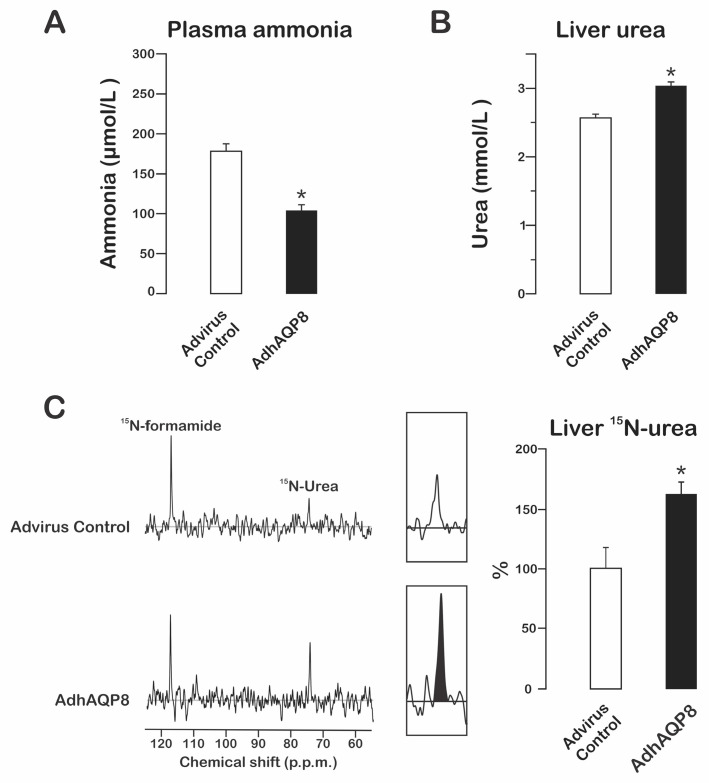
Ammonia-derived ureagenesis in AdhAQP8-transduced normal mice. C57BL/6 mice were transduced with AdhAQP8 (black bars) or a control adenovector (white bars), and ammonium acetate was i.p. injected at 72 h later. (**A**) Plasma ammonia after 15 min of ammonia administration (see Materials and Methods for details). The data are expressed as the means ± SEM of four independent experiments. * *p* < 0.05 from the control. (**B**) Liver urea after 15 min of ammonia administration (see Materials and Methods for details). The data are expressed as the means ± SEM of four independent experiments. * *p* < 0.05 from the control. (**C**) **Left**: representative ^15^N-NMR spectra for urea. **Right**: quantitation of ^15^N-labeled urea normalized to the internal standard, corresponding to four independent ^15^N-NMR spectra. The data are expressed as % of controls and represent the means ± SEM. * *p* < 0.05. NMR, nuclear magnetic resonance.

To elucidate the effects of hAQP8 expression on mouse liver, we determined the ammonia levels in plasma after a single dose of ammonia acetate. The ammonia levels were measured in systemic blood after 15 min of ammonia challenge [20]. The plasma ammonia concentration was significantly lower in the AdhAQP8-transduced mice (Figure 2A). Consistently, the urea content in the liver was found to be increased (Figure 2B). This suggests that ureagenesis is the pathway that controls excess ammonia in the hAQP8-transduced mice. We confirmed this assumption using ^15^N-labeled ammonia and evaluating hepatic ^15^N-labeled urea production. As shown in Figure 2C, hepatic ^15^N-labled ureagenesis is markedly increased in the hAQP8-transduced mice.

Our previous studies using cultured hepatocytes have indicated that gene silencing of endogenous mitochondrial AQP8 or overexpression of mitochondrial AQP8 induces a decrease or an increase in ureagenesis, respectively, but only from free ammonia, and not from amino acids that represent intramitochondrial sources of ammonia [8,9]. Studies using isolated mitochondria that expressed hAQP8 directly confirmed this statement [9]. Thus, the present data from the in vivo studies, together with data obtained from cells and mitochondria [8,9], provide additional support for a key role of AQP8 in mitochondrial diffusional uptake of ammonia and, consequently, its conversion to urea. Furthermore, the fact that mitochondrial hAQP8 expression can increase ammonia-derived ureagenesis in mice suggests that ammonia transport across mitochondrial membranes limits the efficiency of ammonia detoxification from liver cells.

### 3.3. Hepatic Gene Transfer of hAQP8 in Mice with Defective Ammonia-Derived Ureagenesis

**Figure 3:**
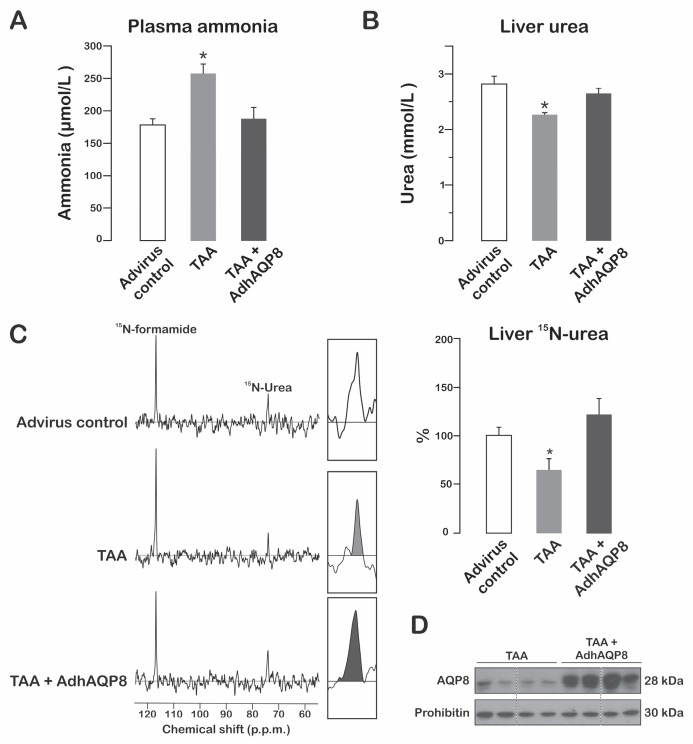
Effect of hAQP8 hepatic gene transfer on TAA-impaired ammonia-derived ureagenesis. Mice were transduced with AdhAQP8 or control adenovector and treated with TAA as detailed in Materials and Methods. (**A**) Plasma ammonia after 15 min of ammonia administration. The data are expressed as means ± SEM of four independent experiments. * *p* < 0.05 from the controls or TAA + AdhAQP8. (**B**) Liver urea after 15 min of ammonia administration (see Materials and Methods for details). The data are expressed as the means ± SEM of four independent experiments. * *p* < 0.05 from the controls or TAA + AdhAQP8. (**C**) **Left**: representative ^15^N-NMR spectra for urea. **Right**: quantitation of ^15^N-labeled urea normalized to the internal standard, corresponding to four independent ^15^N-NMR spectra. The data are expressed as % of the controls and represent the means ± SEM. * *p* < 0.05 from the controls or TAA + AdhAQP8. (**D**) Immunoblotting for AQP8: a 28 kDa immunoreactive band corresponding to endogenous mitochondrial mouse AQP8 and hAQP8 is observed. The AQP8 antibody used is able to detect both mouse and human isoforms but with a higher affinity for the latter. Each lane was loaded with 25 μg of protein. Prohibitin, an inner mitochondrial membrane marker, is shown as the control for equal protein loading.

As our above-mentioned findings indicate that mitochondrially expressed hAQP8 enhances hepatic ammonia-derived ureagenesis in normal mice, we evaluated whether AdhAQP8 transduction could ameliorate defective urea synthesis. Ureagenesis is a metabolic pathway known to be affected by hepatotoxic agents [21]. TAA is a well-established hepatotoxic compound that is used as a model for acute liver toxicity leading to a state of hyperammonemia [22,23]. As shown in Figure 3A,B, the TAA-treated mice exhibit a rise in plasma ammonia levels (about 50%) and an expected decrease in hepatic urea content. The AdhAQP8-transduced TAA-treated mice show normal plasma ammonemia and hepatic urea. The NMR studies using ^15^N-ammonia confirmed the complete recovery of TAA-impaired ammonia-derived ureagenesis in the AdhAQP8-transduced mice (Figure 3C). The transduction of hAQP8 gene and its mitochondrial expression were not affected by TAA treatment to the mice (Figure 3D).

TAA hepatotoxicity is thought to be mainly due to its activation by reactive metabolites, which, in turn, leads to the production of reactive oxygen species and oxidative stress [21,24]. Although the precise mechanisms for TAA-induced reduction in ammonia-derived ureagenesis are still unknown, alterations in the synthesis or activity of urea cycle enzymes and perhaps mitochondrial AQP8 should certainly be involved [11,24]. We believe that hAQP8-induced restoration of mitochondrial ammonia supply is a key factor contributing to the amelioration of defective ammonia-to-urea detoxification caused by TAA.

In conclusion, our data suggest that mice transduced with the adenovector AdhAQP8 can express hAQP8 in hepatocyte mitochondria, which, in turn, improves the conversion of ammonia to urea in normal and defective ureagenesis. These in vivo findings, along with our previous findings using cultured hepatocytes [9], support a key role of mitochondrial AQP8 in facilitating ammonia diffusion for urea synthesis. In addition, hepatic AQP8 gene transfer may have potential therapeutic implications for conditions involving hyperammonemia.

## Data Availability

The data presented in this study are available in the Supplementary Materials.

## Supplementary Materials

The following supporting information can be downloaded at https://www.mdpi.com/article/10.3390/cells12111535/s1. Figure S1: Mitochondrial hAQP8 expression in mice.
