# Supplementary material for: Adenoviral Transfer of Human Aquaporin-8 Gene to Mouse Liver Improves Ammonia-Derived Ureagenesis
Source: Cells. 2023 Jun 2;12(11):1535. doi: 10.3390/cells12111535 (PMC10253139; doi:10.3390/cells12111535)
Supplement: Supplementary file 1 [file cells-12-01535-s001.zip › Suppl.pdf]

Supplementary Information

Adenoviral transfer of human aquaporin-8 gene to mouse liver improves ammonia-derived ureagenesis

Alejo M. Capiglioni, María C. Capitani, Julieta Marrone and Raúl A. Marinelli

Correspondence: rmarinel@unr.edu.ar; marinelli@ifise-conicet.gov.ar

SUPPLEMENTARY FIGURES

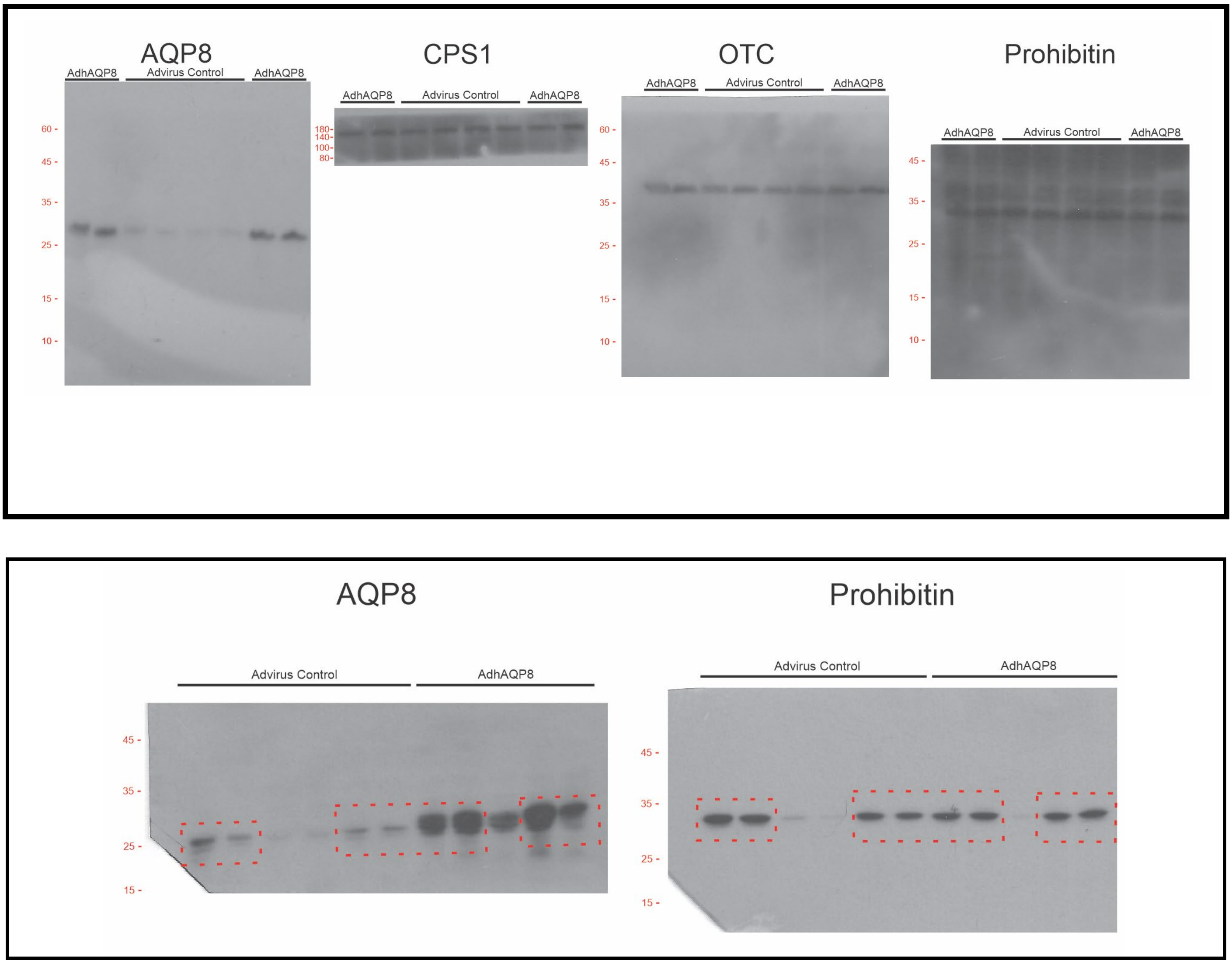

Figure S1. Mitochondrial hAQP8 expression in mice. Uncropped blot lanes
